# Preoperative CT Evaluation of Abdominal Vasculature and the Risk of Surgical Complications in Colorectal Cancer Resection with Anastomosis: A Systematic Review and Meta-Analysis

**DOI:** 10.3390/diagnostics16101449

**Published:** 2026-05-09

**Authors:** Mihnea-Ionuț Nicoară, Mihai Adrian Socaciu, Diana Ursu, Andra Ciocan, Nadim Al Hajjar

**Affiliations:** 1 Department of Surgery—Surgery III, “Iuliu Hațieganu” University of Medicine and Pharmacy, 400012 Cluj-Napoca, Romania; mihnea.ionu.nicoara@elearn.umfcluj.ro (M.-I.N.); andra.ciocan@umfcluj.ro (A.C.); nadim.alhajjar@umfcluj.ro (N.A.H.); 2 Department of Radiology, Emergency Clinical County Hospital Cluj-Napoca, 400006 Cluj-Napoca, Romania; dia.ursu@elearn.umfcluj.ro; 3 Department of Medical Imaging and Nuclear Medicine, “Iuliu Hațieganu” University of Medicine and Pharmacy, 400012 Cluj-Napoca, Romania; 4 Department of Medical Imaging, “Octavian Fodor” Regional Institute of Gastroenterology and Hepatology, 400162 Cluj-Napoca, Romania; 5 Department of Radiology, “Iuliu Hațieganu” University of Medicine and Pharmacy, 400012 Cluj-Napoca, Romania; 6 Department of Surgery, “Octavian Fodor” Regional Institute of Gastroenterology and Hepatology, 400162 Cluj-Napoca, Romania

**Keywords:** computed tomography, vascular imaging, vascular calcification, mesenteric stenosis, colorectal cancer, colorectal surgery, post-operative complications, anastomotic leak, systematic review, meta-analysis

## Abstract

**Background**: Preoperative CT is part of the routine diagnostic work-up for colorectal cancer (CRC), and CT-based biomarkers have been linked to oncological and surgical outcomes in CRC patients. This review aims to evaluate the association between preoperative CT-derived vascular disease markers (calcification and stenosis) and postoperative outcomes after curative CRC resection with anastomosis. **Methods**: Following PRISMA, we conducted a systematic review and meta-analysis of studies identified from MEDLINE/PubMed, Embase, Web of Science Core Collection and Google Scholar from inception until 1st of January 2026, with the protocol registered in PROSPERO (CRD420251248044). Eligible studies examined CT-derived abdominal vascular disease markers in CRC patients treated with curative resection and anastomosis and reported postoperative outcomes (anastomotic leakage (AL) grade C, major morbidity, and mortality). Risk of bias was assessed using the Newcastle–Ottawa Scale. We pooled odds ratios using random-effects models when ≥3 studies reported comparable exposure–outcome comparisons. **Results**: Fourteen studies (6712 participants) were included, and 12 contributed to quantitative synthesis. Higher calcification burden was associated with increased odds of any AL (OR 3.08, 95% CI 2.09–4.54; *k* = 11; *n* = 5005) and severe/grade C AL (OR 2.68, 95% CI 1.03–6.97; *k* = 3; *n* = 3418). Evidence for major morbidity was imprecise (OR 1.99, 95% CI 0.86–4.59; *k* = 3; *n* = 841), and data for mesenteric stenosis outcomes and mortality were limited. Sensitivity analyses indicate attenuation without loss of significance after trim-and-fill (adjusted OR 2.39) and that non-ROC cut points yield a smaller effect size (OR 2.42). **Conclusions**: CT-derived vascular disease markers are associated with higher odds of AL after CRC surgery. Prospective studies should standardize methods and test clinical utility.

## 1. Introduction

Colorectal cancer (CRC) is the third most common type of cancer and the second leading cause of cancer death worldwide, according to GLOBOCAN 2022 [1]. Surgical resection is central to curative treatment for both colon and rectal cancer and it commonly involves primary anastomosis to restore bowel continuity [2,3]. The ESCP CORREA 2022 snapshot audit [4], which enrolled 3521 adults undergoing colorectal resection across 216 hospitals in 53 countries during a 6-week period (January to April 2022) captured early (30-day) postoperative outcomes. The Clavien–Dindo (C-D) classification is the generally accepted system used for reporting complications after surgery; grades are assigned by the required therapy, from minor deviations (grade I) through complications requiring surgical, endoscopic or radiological intervention (grade III, with IIIa without and IIIb with general anesthesia), life-threatening complications requiring intensive care (grade IV) and death (grade V) [5]. Based on this, CORREA defined short-term morbidity as the worst C-D grade within 30 days and reported that 47% of patients had no recorded 30-day complications, while severe complications (C-D grade III–V) occurred in 12.2% of patients; the 30-day mortality rate was 2.38% [4]. Among complications, anastomotic leakage (AL) is especially relevant as it can progress to peritonitis and severe sepsis, requiring urgent intervention. The International Study Group of Rectal Cancer defines AL as a defect of the intestinal wall at the anastomotic site causing a communication between the intra- and extraluminal compartments and grades severity according to the corresponding change required in clinical management (grade A, no change in management; grade B, active therapeutic intervention without relaparotomy; grade C, relaparotomy) [6]; in CORREA, AL occurred within 30 days in 7.96% of cases [4]. Leakage has been associated with substantial short-term morbidity and hospital mortality. For example, in a German nationwide quality assurance cohort of colon cancer resections, secondary complications occurred in 62.7% of patients with leakage versus 19.9% without leakage and hospital mortality was 18.6% versus 2.6% [7]. Moreover, leakage may also compromise long-term oncologic outcomes, including increased local recurrence risk and reduced survival [8,9]. According to economic analyses, AL generates significant incremental healthcare costs on account of readmissions, intensive care stay and reinterventions [10].

Contrast-enhanced CT (CECT) is routinely recommended for pretherapeutic staging of colorectal cancer, primarily to identify distant metastatic disease and other findings that may alter oncological treatment and/or surgical resectability [11,12]. Recent quantitative imaging work has examined several CT-derived biomarkers with potential applications in oncology, which can be broadly grouped into two categories: tumor-focused and systemic ones. The first category quantifies imaging phenotypes related to tumor heterogeneity and the tumor microenvironment; a systematic review of colorectal cancer radiomics in CT, MRI and PET summarized evidence on prediction of treatment response and survival, showed promising results, but significant methodological heterogeneity [13]. Meanwhile, systemic imaging biomarkers such as CT-derived body composition metrics (e.g., quantification of skeletal muscle and adipose compartments) have also been evaluated for their ability to predict both long-term oncological outcomes [14] and short-term postoperative complications, including those arising after CRC surgery [15]. A less explored category of systemic CT biomarkers evaluates vascular disease using atherosclerosis markers such as calcification burden and splanchnic arterial stenosis as potential predictors of anastomotic leakage and other early postoperative outcomes after colorectal resection, with conflicting findings across cohorts [16,17,18,19,20,21]. Because CECT is already part of the standard pretherapeutic staging in colorectal cancer, CT-derived vascular findings can be regarded as opportunistic biomarkers extractable from routine imaging rather than additional dedicated tests. Full diagnostic assessment nevertheless extends beyond CT and, particularly in rectal cancer, includes histologic confirmation, dedicated locoregional staging and multidisciplinary treatment planning. Beyond routine staging CT, interest is also growing in vascular-focused imaging approaches across the perioperative pathway, including indocyanine green fluorescence angiography (ICG-FA) methods that may facilitate identification of the inferior mesenteric artery [22].

The purpose of this study is to integrate the evidence on the association between preoperative CT-derived abdominal vascular disease markers, including calcification burden and mesenteric arterial stenosis and early postoperative outcomes after colorectal cancer surgery with primary anastomosis, focusing on overall anastomotic leakage, clinically impactful leakage, major morbidity and short-term mortality. Effect measurements were pooled according to biomarker families for the prespecified outcomes, and the certainty of evidence was rated using the GRADE framework.

## 2. Methods

The present study was conducted in accordance with the PRISMA guidelines [23] and the study protocol was prospectively registered in the PROSPERO database, with the reference number CRD420251248044 (https://www.crd.york.ac.uk/prospero, accessed on 11 December 2025). No amendments to the study protocol were made after registration.

### 2.1. Search Strategy

We searched PubMed, Embase, Web of Science Core Collection and the first 200 results of targeted Google Scholar search queries from inception to 1 January 2026. The following index terms and free-text words were used, including synonyms and sub-categories, grouped into blocks following the patient, exposure, outcome (PEO) framework: “colorectal neoplasms”, “colorectal surgery”, “diagnostic imaging” and “postoperative complications”. Reference lists of included studies were also screened. Only English-language results were screened because of resource limitations. The most recent search was conducted on 1 January 2026. Search strings were designed using PubMed syntax and translated for use on Embase and WoS Core Collection using the Polyglot Search Translator (https://polyglot.sr-accelerator.com [accessed on 11 December 2025]) [24]. Full search strings are provided in Appendix A and the Supplementary Materials.

### 2.2. Study Selection

Search results were collected and organized using the reference management software Zotero (https://www.zotero.org). Duplicates were automatically flagged using Zotero’s built-in features and manually removed before screening. Two reviewers (M.N. and D.U.) independently screened titles/abstracts and excluded reports using the following criteria: (1) no full text was available, (2) full text was not translated into English, (3) reviews, editorials, conference abstracts, proceedings, videos, letters to the editor and comments, (4) animal studies, (5) study participants <18 years of age, (6) surgery for benign pathologies, (7) studies exclusively on intraoperative vascular assessment, (8) postsurgical anastomotic complications were not reported as an outcome. Full-text articles were included if (1) study participants were surgically treated for colon and/or rectal neoplasms with curative intent, (2) CT was performed before surgery, (3) abdominal vascular disease (calcification burden or arterial steno-occlusive disease) was evaluated on preoperative CT, (4) outcomes included prediction of post-surgical complications. In the case where a consensus was not achieved, a third reviewer (M.S.) was called to arbitrate.

Studies enrolling mixed benign and malignant indications were eligible only if CRC-only data were reported or could be obtained. For mixed-indication cohorts without CRC-stratified outcomes, we contacted corresponding authors to request CRC-only aggregate subgroup counts.

### 2.3. Data Extraction

Two independent reviewers (M.N. and D.U.) extracted study design/setting, patient characteristics (age, sex, tumor site, and procedure), CT exposure definitions for calcification and stenosis (territory, scoring method, and threshold) and raw count 2 × 2 tables for each exposure–outcome comparison. For quantitative synthesis, primary pooled estimates were based on crude, study-level 2 × 2 exposure–outcome data after harmonization of high vs. low vascular disease categories. Adjusted estimates were extracted when available and tabulated separately but were not pooled quantitatively. Primary outcomes were major morbidity (Clavien–Dindo ≥III), anastomotic leakage (overall and/or grade C), and postoperative mortality as reported by each study, including the stated follow-up window. No automation tools were used for data extraction.

For meta-analysis, we categorized vascular markers as high vs. low using the threshold reported in each study’s primary analysis. When several thresholds were reported, we prioritized clinically relevant distinctions (i.e., moderate vs. severe calcific burden or stenosis), or, if not possible, we used the authors’ principal cut-off and recorded how it was chosen (prespecified, median or ROC-derived). To account for possible information loss due to dichotomization of continuous variables and ROC-derived cut-off optimism, we applied sensitivity analyses by threshold type (including analyses excluding ROC-derived cut-offs). When available, we also extracted continuous effect estimates for separate (non-pooled) analyses. For multiple vascular territories or composite scores, we prioritized broader coverage, then proximity to the anastomosis.

### 2.4. Bias and Certainty Assessment

Two reviewers (M.N. and M.S.) assessed risk of bias using the Newcastle–Ottawa scale (NOS), with disagreements adjudicated by a third reviewer (N.A.H.). Studies were classified as being of poor, fair or good quality using scores of 0–3, 4–6 and 7–9, respectively.

Certainty of evidence was evaluated based on the GRADE framework [25]. The predefined primary and secondary outcomes were evaluated regarding risk of bias, inconsistency, indirectness, imprecision, and publication bias, contributing to their respective certainty rating.

### 2.5. Statistical Analysis

Analyses were conducted in R version 4.5.2 (RStudio environment, version 2025.09.0-387), using the meta and metafor packages. Each study contributed a single effect estimate to any given pooled comparison. Studies with zero events in one exposure arm were retained using a 0.5 continuity correction. Studies with zero events in both exposure arms were prespecified as non-informative for odds ratio pooling and excluded from the corresponding outcome-level analysis. Quantitative synthesis was performed when ≥3 studies reported similar exposure–outcome definitions. Pooled effects were estimated using a random-effects model with inverse-variance weighting and the results are presented using forest plots. Between-study variance (τ^2^) was estimated using restricted maximum likelihood (REML) and random-effects confidence intervals were calculated using the Hartung–Knapp–Sidik–Jonkman (HKSJ) adjustment, consistent with current Cochrane guidance [26]. This approach was preferred to better reflect uncertainty in the pooled estimate and between-study heterogeneity compared with the conventional DerSimonian–Laird random effects inference, particularly important when the number of studies is small [26]. Statistical heterogeneity was summarized using τ^2^ and I^2^ (with ~25%, ~50%, and ~75% indicating low, moderate, and high heterogeneity, respectively). Potential publication bias and small-study effects were assessed by visual inspection of funnel plots, Egger’s regression test (when ≥10 studies were available) and by trim-and-fill analysis. Sensitivity analysis included leave-one-out influence tests and categorical moderator analysis of different harmonization decisions and methodological differences (binary vs. multi-category contrasts, visual vs. quantitative exposure measurement, ROC-derived vs. prespecified cut points, vascular territory and study quality/risk-of-bias category) (Appendix D). In addition, post hoc exploratory univariable meta-regressions were performed for the any AL/AAC synthesis to further explore the sources of heterogeneity using total sample size, scaled per 100 participants, publication year and geographic region (Asia vs. Europe) as covariates. Mixed-indication vascular stenosis studies were analyzed separately as an exploratory post hoc analysis for contextual purposes and were not incorporated into the main CRC-only findings (Appendix E).

## 3. Results

### 3.1. Study Selection

**Figure 1:**
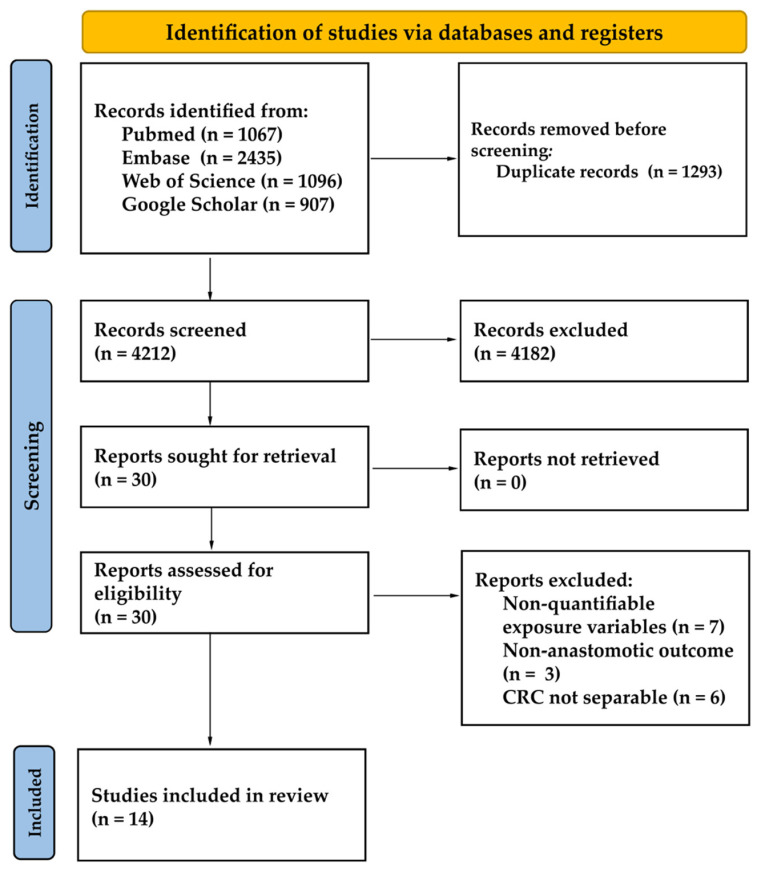
PRISMA 2020 flow diagram for this systematic review.

Initial search and deduplication yielded 4212 records eligible for title and abstract screening, out of which 30 were reviewed by full-text assessment. Overall, 14 studies [19,20,21,27,28,29,30,31,32,33,34,35,36,37] fulfilled the pre-determined inclusion criteria and 12 could be included in the quantitative synthesis. The reasons for exclusion were absence of quantifiable exposure variables (i.e., studies of vascular anatomy, *k* = 7 [38,39,40,41,42,43,44]), exclusively non-anastomotic negative outcomes (high-output ileostomy [45], surgical difficulty [46] and colonic hypoperfusion [47]) and lack of CRC-only subgroup data on outcomes (*k* = 6; [17,18,48,49,50,51]). Of the studies excluded for unavailable CRC-only, four accounted for malignant/benign indication status and included it as a covariate; for these studies CRC-stratified counts were requested from the corresponding authors, but no response was received. The complete study selection algorithm can be consulted in the PRISMA flow diagram (Figure 1).

### 3.2. Characteristics of the Included Studies

Fourteen studies (Table 1) comprising 6712 participants were included in our analysis. Cohort sizes ranged from 60 to 2412 patients. Most studies reported a male predominance (typically 50.0–68.8% male) and the mean/median participant age ranged from 63.7 to 75.3 years, reflecting predominantly older surgical populations.

Methodologically, the evidence base was largely retrospective (*k* = 11) and single-center (*k* = 13). Thresholds used to dichotomize exposure were most commonly ROC/Youden-derived (*k* = 5; [27,30,31,33,37]) and median-based (*k* = 2; [28,35]), while 3 studies applied prespecified ordinal/segment staging [21,34,36]; single studies used an author-defined composite threshold [29], binary presence/absence [20], continuous-only comparisons without a cut point [32] or clinical stenosis grades [19]. Studies reported a follow-up window for outcome detection of 30 days (*k* = 5; [20,21,28,31,33]) or 90 days (*k* = 2; [19,34]).

Exposure measurement was either visual/semi-quantitative (*k* = 6; [20,21,28,29,34,36]), software-derived quantitative (*k* = 7; [27,30,31,32,33,35,37]) or evaluated as stenosis grading [19]. Regarding vascular territory, five studies assessed the abdominal aorta only [21,30,31,35,36], three assessed an aortoiliac territory [28,29,33], two focused on splanchnic arteries only [19,34], three combined aortic and splanchnic territories [20,27,37] and one sampled multiple pelvic vessels plus the superior mesenteric artery (SMA) [32].

Twelve studies contributed data suitable for quantitative synthesis: 11 were included in the meta-analysis of any anastomotic leakage [20,21,28,29,30,31,33,34,35,36,37], and 3 studies contributed to the major morbidity [21,27,28] and grade C anastomotic leakage [20,29,35] analyses each. Two studies were synthesized narratively: Norooz et al. [32] was a comparative cohort study on participants with and without AL that measured differences in mean calcium score, thus precluding effect estimation; Arron et al. [19] was the only CRC study reporting extractable data on abdominal vasculature stenosis measurements and was therefore not pooled.

#### Risk of Bias Assessment

Across 14 studies, seven were rated “Good” and seven were “Fair” on the Newcastle–Ottawa Risk of Bias Scale, with a median score of six points (range 5–8). Selection scores were uniformly high (4/4), cohorts reflected the typical surgical population, vascular exposure status was determined from preoperative CT records and all outcomes were postoperative by definition. Variation was greatest in the comparability domain: four studies reported unadjusted comparisons (0/2), seven applied limited confounder control (limited adjustment or matching; 1/2), while three studies applied multivariable adjustment (2/2). Only Eveno et al. [21] achieved 3/3 on the “Outcome” domain because outcome ascertainment, follow-up duration, and follow-up completeness were all explicitly reported. While the other studies had objective outcome assessment, incomplete reporting of follow-up completeness and/or the postoperative follow-up window warranted downgrade.

A summary of NOS scores is provided in Table 1, with item-level assessments available in Appendix B. Reported adjusted and matched estimates, along with the variables or matching factors used in each study, are summarized in Appendix C.

### 3.3. Summary of Findings

Using the GRADE framework, the certainty of evidence for each of the predefined critical outcomes was evaluated, with a summary available in Table 2. We reported the pooled random-effects OR from the meta-analysis and its 95% CI, the number of participants contributing to the estimate and an absolute risk estimate derived from baseline event rates in the low-vascular-disease group and expected risk in patients with substantial vascular disease. Certainty ratings reflect domains of risk of bias, inconsistency, indirectness, imprecision and publication bias. Observational evidence was initially rated as low certainty. Upgrading was considered for a large magnitude of effect. Sensitivity analyses were considered when judging inconsistency and imprecision but were not treated as an independent upgrading domain.

The overall certainty of evidence ranged from “low” (for the AAC score—any anastomotic leak exposure-outcome pair) to “very low” (the other two AAC score exposure-outcome pairs and outcomes related to stenosis and mortality). The chief reason for downgrade was concern regarding the risk of bias, stemming from the predominantly retrospective and non-randomized study designs. Table 2 summarizes the main findings, and the full domain-level GRADE decision process is provided in Appendix F.

### 3.4. Relationship Between Calcium Score and Surgical Complications

**Figure 2:**
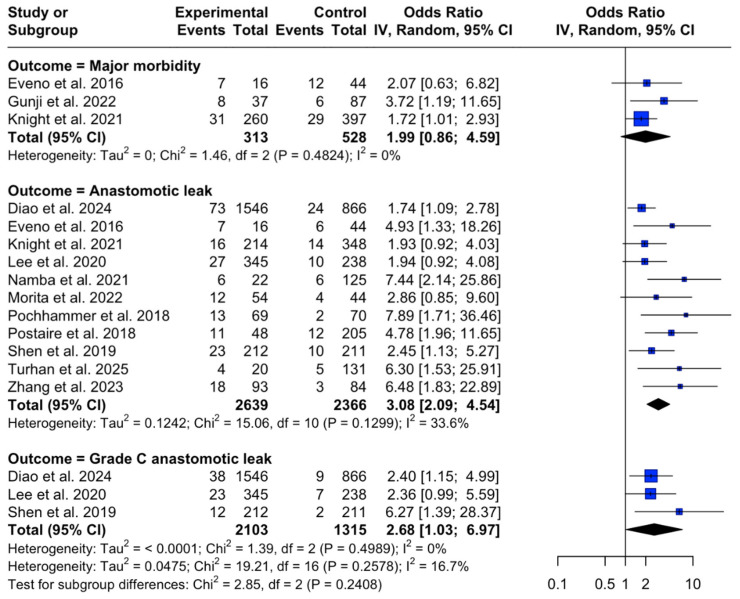
Forest plot showing the odds ratios (ORs) and 95% confidence intervals (CIs) of abdominal CT calcification scores for the prediction of postoperative complications. Outcomes are grouped into “Major morbidity”, “Anastomotic leak” and “Grade C anastomotic leak” and only subgroup-level aggregate statistics are reported. Squares represent individual study effects (size proportional to study weight) and diamonds represent pooled effects; horizontal lines indicate 95% CIs. No double-zero event study–outcome comparisons were excluded from the pooled calcification analyses shown; the exclusion rule was prespecified but not triggered [20,21,27,28,29,30,31,33,34,35,36,37].

Major morbidity (*k* = 3; OR 1.99, 95% CI 0.86–4.59; I^2^ = 0%; Figure 2): Eveno et al. [21] used a simple abdominal aortic calcification score (ACS) based on circumferential involvement (<50% vs. >50%) between the celiac trunk origin and aortic bifurcation and reported higher rates of severe postoperative events with increasing ACS, including more clinically significant leaks requiring reintervention. Gunji et al. [27] quantified an Agatston-type calcium score around the SMA/IMA origins and defined major morbidity as Clavien–Dindo ≥III; an optimal AAC cut-off of 10 identified patients with markedly higher complication rates, particularly in conjunction with abnormal cardio-ankle vascular index (CAVI). Knight et al. [28] (largest cohort, *n* = 657) used a semi-quantitative visual aortic score (none/minor/major) at the proximal and distal level and found that higher burden was associated with non-infective complications.

Any anastomotic leak (*k* = 11; OR 3.08, 95% CI 2.09–4.54; I^2^ = 33.6%; Figure 2): Most studies showed higher leak risk with greater calcification, but effect sizes varied with scoring methods, cut-offs and population. Larger cohorts (Diao et al. [20] *n* = 2412; Knight et al. [28]) reported weaker and, in the case of Diao, non-independent effect after multivariable adjustment. In contrast, selected cohorts showed stronger gradients: Eveno et al. [21] showed an increase in AL risk across ACS categories; Morita et al. [30] quantified calcification volume percentages using dedicated software (median 2.35%, range 0–40.3%) and Shen et al. [35] derived a custom aortoiliac calcification index (ACI) using a 12-sector method (median 4.8%, range 0–91.1%)—both studies reporting independent associations with AL; the largest effect size was observed by Namba et al. [31] in a cohort of 147 participants, using an Agatston-type measurement and multivariate logistic regression (OR = 6.09 after adjustment, with a 95% CI of 1.48–25.86 for calcium score cut point of 4007). Lee et al. [29] used a pragmatic aorto-iliac score (three-level semi-quantitative assessment of infrarenal aorta and common iliac calcification) and reported borderline association with AL in general, but found that a score ≥ 3 more clearly predicted severe leaks. Norooz et al. [32] found higher calcium scores in the descending aorta and all great pelvic vessels among patients with AL (20% AL incidence in cohort; AAC score of 792 ± 39 vs. 405 ± 45 in AL vs. non-AL patients, respectively; significant at *p* < 0.001), but could not be pooled because results were reported as continuous scores rather than dichotomized groups. Very large effects were reported in some specialized scoring approaches reporting various stratification systems and cut-off values, likely contributing to heterogeneity: Pochhammer et al. [33] (infrarenal aorta and iliac-focused blinded clinical trial; iliac volume score ≥30 as cut-off value), Postaire et al. [34] (visceral arterial calcification score ≥3 and celiac trunk score = 2), Turhan et al. [36] (celiac-to-bifurcation aortic circumference staging; stage 2 ≥50%), Zhang et al. [37] (dedicated CT calcium scoring workflow at SMA and IMA origin; the AAC score cut-off of 19.6 mm^3^ that was incorporated in a nomogram). Both Turhan and Zhang reported independent associations after adjustment, whereas Pochhammer acknowledged residual confounding, as renal disease remained the only independent predictor in multivariable modeling.

Grade C anastomotic leak (*k* = 3; OR 2.68, 95% CI 1.03–6.97; I^2^ = 0%; Figure 2): Lee et al. [29] found that an aorto-iliac score ≥ 3 independently predicted grade C AL. Shen et al. [35] reported higher ACI with increasing AL severity. Diao et al. [20] observed higher grade C rates with AAC but no independent association following multivariate adjustment.

#### 3.4.1. Sensitivity Analysis

**Figure 3:**
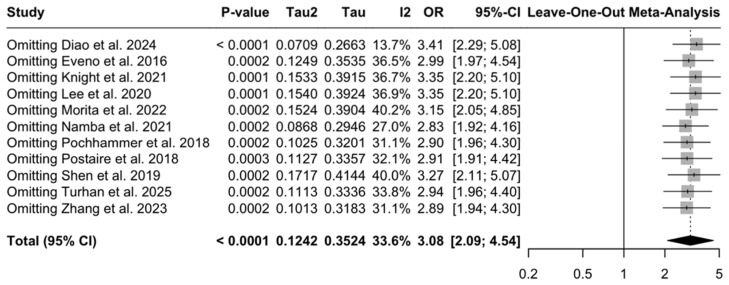
Leave-one-out influence analysis for the random-effects meta-analysis of high vs. low CT calcium score and any anastomotic leak. Each row shows the pooled odds ratio and heterogeneity estimates after omitting the named study [20,21,28,29,30,31,33,34,35,36,37].

**Figure 4:**
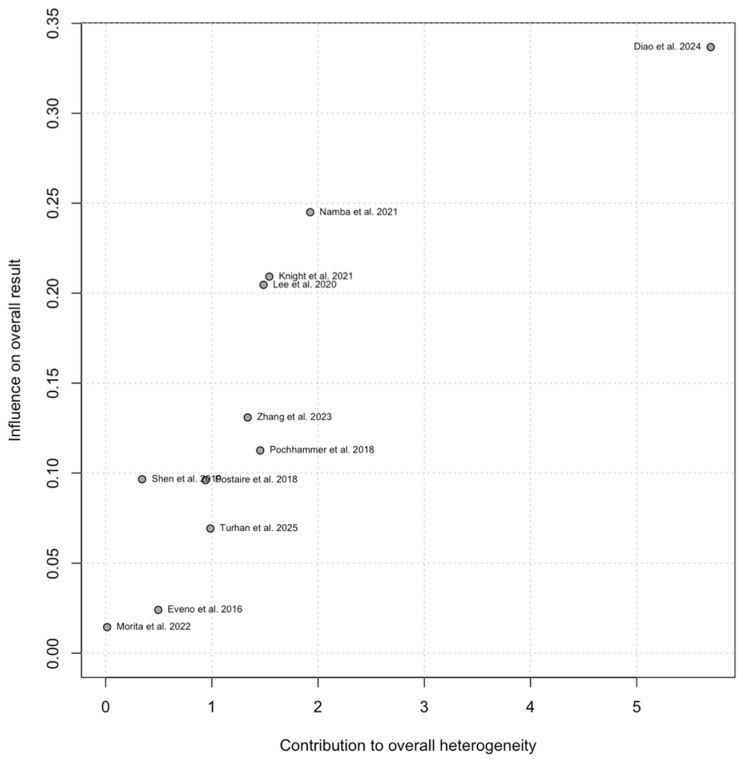
Baujat plot for the random-effects meta-analysis of AAC and any AL. The x-axis shows each study’s contribution to overall heterogeneity, and the y-axis shows its influence on the pooled effect estimate [20,21,28,29,30,31,33,34,35,36,37].

Influence diagnostics using leave-one-out analysis for any AL/AAC score showed that the pooled association was stable and not driven by any single study (Figure 3). For each omission, pooled ORs ranged from 2.83 to 3.41 and remained statistically significant (*p* < 0.0001). The largest shift occurred when Diao et al. [20] was omitted, with an associated reduction in heterogeneity (I^2^ = 13.7%), indicating that this study was the largest single contributor to between-study variability. Other plausible sources of heterogeneity include differences in study size, vascular territory, exposure measurement approach, threshold derivation and outcome definitions. Publication year and geographic region may also act as proxies for underlying study-level differences contributing to between-study variability. A post hoc exploratory univariable meta-regression was therefore performed for the any AL/AAC synthesis to further examine potential sources of heterogeneity (Table A7). Larger study sample size was associated with smaller observed effect estimates, suggesting potential small-study effects, whereas publication year and geographic region showed no clear association with effect size. Omitting Pochhammer et al. [33] (the only study in the any-AL subgroup with prospective data acquisition) did not significantly influence the pooled effect (random-effects OR 2.90, 95% CI 1.96–4.29; *p* < 0.0001) and heterogeneity remained moderate (τ^2^ = 0.10; I^2^ = 31.1%). A Baujat plot (Figure 4) also indicated that Diao et al. 2024 [20] contributed most to between-study heterogeneity and had the greatest influence on the pooled estimate. In general, heterogeneity remained low (I^2^ ~13.7–40.2%, τ^2^ ~0.07–0.17), indicating that the overall conclusion was robust to single-study exclusion.

**Figure 5:**
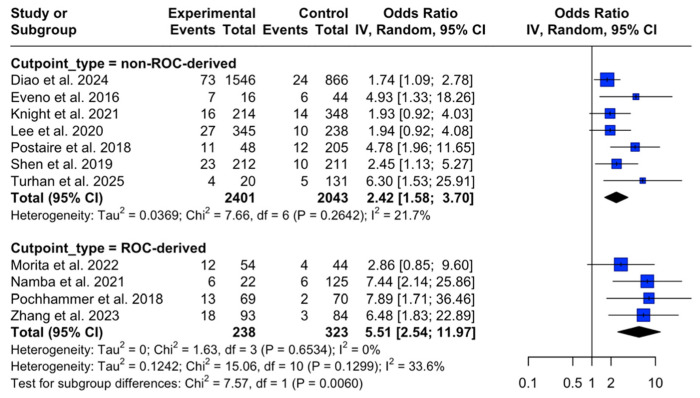
Forest plot of the subgroup meta-analysis for the association between AAC score and any AL, stratified by calcification cut point (non-ROC-derived vs. ROC-derived) [20,21,28,29,30,31,33,34,35,36,37]. Squares represent individual study effects (size proportional to study weight) and diamonds represent pooled effects; horizontal lines indicate 95% CIs.

In categorical moderator analyses (Table A5, Appendix D), we explored whether the association between higher vascular disease burden and any anastomotic leak varied by the following study characteristics: cut point derivation, exposure measurement approach, vascular territory, and risk of bias/NOS grade. Studies using ROC-derived thresholds showed a larger pooled effect (*k* = 4; OR 5.51, 95% CI 2.54–11.97; I^2^ = 0%) and this difference was statistically significant (F = 5.42, *p* = 0.044; ratio of ORs 2.32, 95% CI 1.02–5.24), consistent with ROC cut point optimism. After excluding studies that reported ROC-derived cut points, the association between higher calcification burden and any anastomotic leak persisted in the non-ROC-derived group (*k* = 7; random-effects OR 2.42, 95% CI 1.58–3.70; I^2^ = 21.7) (Figure 5). No statistically significant effect modification was detected for the other moderators (exposure measurement approach, vascular territory, or NOS grade), supporting the robustness of the pooled association to the main harmonization assumptions.

A post hoc comparison with conventional DerSimonian–Laird random effects inference resulted in narrower confidence intervals than the primary REML + Hartung–Knapp approach. The direction of effect was unchanged for all pooled outcomes. Major morbidity estimates changed nominal statistical significance, indicating estimator sensitivity in a sparse, three-study synthesis.

#### 3.4.2. Publication Bias

**Figure 6:**
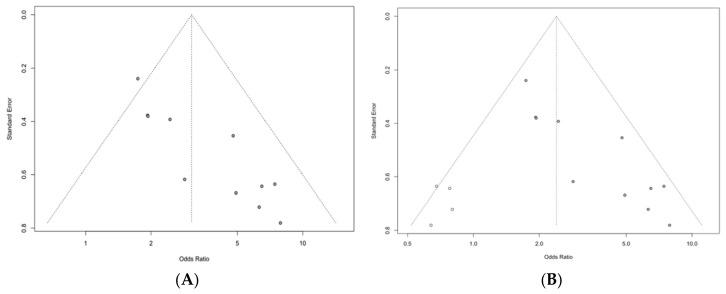
Funnel plot for any AL. (**A**) Observed funnel plot. (**B**) Funnel plot after trim-and-fill (imputed studies shown as open points).

Visual interpretation of the funnel plot (Figure 6) indicated asymmetry, which was supported by Egger’s regression test (t = 5.93, df = 9, *p* = 0.0002). Using the trim-and-fill method, four studies were imputed, increasing the analysis from 11 observed to 15 studies. The bias-adjusted random-effects model yielded a reduced, but still significant association between higher AAC and any anastomotic leak (OR 2.39, 95% CI 1.53–3.75; *p* = 0.0009). Residual heterogeneity remained substantial (τ^2^ = 0.24; I^2^ = 49.2%; Q = 24.57, *p* = 0.0162).

### 3.5. Relationship Between Abdominal Vessel Stenosis and Surgical Complications

Only Arron et al. [19] met the CRC-specific eligibility criteria for mesenteric arterial stenosis. In this retrospective multicenter cohort study (*n* = 1273) with a blinded, nested case–control design (52 clinically relevant anastomotic leaks vs. 52 controls; matched for age, BMI and cardiovascular comorbidity) evaluating mesenteric occlusive disease on preoperative contrast-enhanced CT, clinically relevant anastomotic leak was defined as requiring reintervention within 90 days and arterial stenoses (inferior mesenteric, superior mesenteric and celiac) were graded using standard severity categories (<50%, ≥50% and ≥70%). Severe IMA stenosis/occlusion (≥70–100%) was significantly more frequent in leak cases than controls (21.2% vs. 1.9%; *p* = 0.01), whereas SMA and celiac stenosis did not differ between groups. Because no other CRC-specific stenosis study provided extractable effect estimates, no CRC-only quantitative synthesis was performed for stenosis. A post hoc exploratory contextual summary of mixed-indication stenosis studies (*k* = 3) is provided in Appendix E; these studies were not included in the main CRC-specific evidence synthesis, GRADE certainty assessment or conclusions.

### 3.6. Relationship Between Abdominal Vascular Disease and Mortality

Mortality was infrequently reported and inconsistently defined; therefore, quantitative synthesis was not performed. Vascular exposure mortality information was available from two AAC studies. Eveno et al. [21] reported three postoperative deaths, all in the severe calcification group within 30 days after surgery, whereas Gunji et al. [27] reported no mortality. Additional deaths were reported by Norooz et al. [32] (1/100; death due to AL at 2 weeks) and Shen et al. [35] (one in-hospital death in the AL group and one in the non-AL group), but these were not stratified by calcium score category and therefore could not contribute to vascular exposure mortality assessment. Arron et al. [19] reported four deaths within the first year after surgical intervention, but perioperative timing and stenosis status were not provided. Consequently, mortality was summarized as a narrative outcome and rated as very low-certainty evidence in the GRADE assessment.

## 4. Discussion

**Figure 7:**
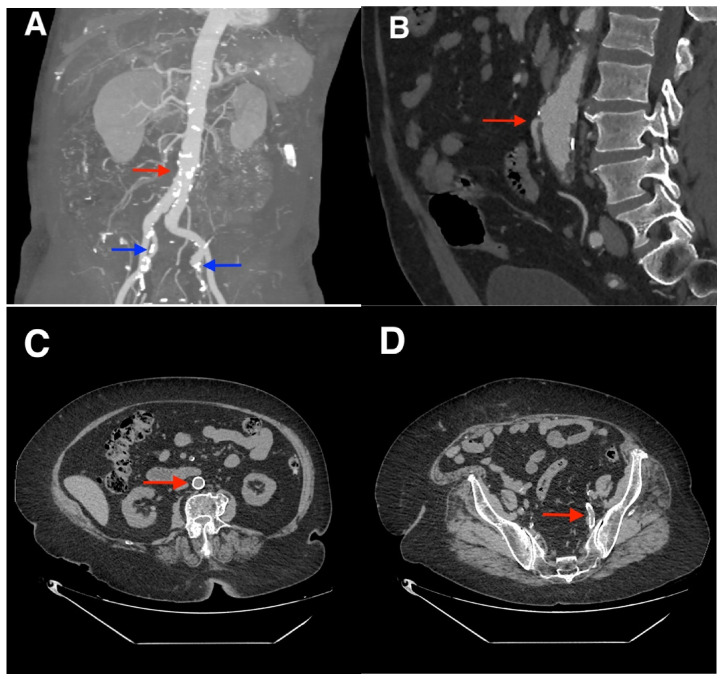
Examples of CT-derived vascular disease markers. (**A**) 3D maximum intensity projection reconstruction of the abdominal vessels showing significant calcific burden predominantly concentrated in the infrarenal abdominal aorta (red arrow) and the internal iliac arteries (blue arrows). (**B**) CT angiography showing stenosis of the inferior mesenteric artery (red arrow). (**C**) Non-contrast CT image at the L3 vertebral level showing calcifications covering the entire circumference of the inferior abdominal aorta (red arrow). (**D**) Same examination: the red arrow points to calcifications involving the internal iliac artery.

In this review, we explored the association between preoperative CT-derived vascular atherosclerosis metrics and adverse postoperative outcomes after elective colorectal cancer resection with primary anastomosis. The two exposure categories evaluated in our study were calcification burden and mesenteric/visceral arterial stenosis indices. Results could be pooled for three of the prespecified outcomes, namely overall anastomotic leakage, clinically severe leakage requiring reoperation and major postoperative morbidity, whereas short-term mortality and stenosis-based outcomes could not be synthesized with comparable confidence, because reporting was sparse and heterogeneous. Across pooled analyses, higher calcification burden was associated with increased odds of anastomotic leakage and severe leak (any AL: OR 3.08; 95% CI 2.09–4.54; *k* = 11, Figure 2; grade C AL: OR 2.68; 95% CI 1.03–6.97; *k* = 3, Figure 2). Major morbidity was not significant in the primary REML + Hartung–Knapp analysis. Although the post hoc DerSimonian–Laird comparison (Table A6) produced narrower CIs and crossed the threshold for nominal statistical significance, this did not alter the interpretation given the small body of evidence (*k* = 3). We therefore retained major morbidity as imprecise and hypothesis-generating, while the association between calcification burden and any anastomotic leak remained the principal stable pooled finding. We emphasize that the observed associations should be interpreted as risk marker relationships rather than practical thresholds for clinical decision-making (e.g., diversion) to be used in isolation. Although calcification burden was the most consistently supported CT-derived vascular marker across included studies, the currently available evidence for mesenteric stenosis, particularly IMA stenosis/occlusion, remains limited and does not support a definitive hierarchy of prognostic importance. Figure 7 provides examples of the CT-derived vascular disease markers explored.

Sensitivity testing did not significantly change the pooled estimates. Leave-one-out analyses preserved the direction and statistical significance of the associations, suggesting that the overall effects were not due to the influence of any individual study (leave-one-out pooled OR of 3.08, range 2.83–3.41; Figure 3). Trim-and-fill decreased the pooled association from OR 3.08 to OR 2.39 (95% CI 1.53–3.75), imputing four studies without changing direction (Figure 6), potentially indicative of small-study effects. Between-study variability of the “any AL” subgroup was moderate (I^2^ = 33.6%; τ^2^ = 0.12; Figure 2) and appeared partly as a result of exposure definition differences, with larger effects observed in studies using ROC/Youden-derived thresholds (Figure 5). Although influence diagnostics identified Diao et al. [20] as the largest contributor to between-study variability, exploratory post hoc meta-regression suggested that study size was also inversely associated with effect magnitude. Together with the funnel plot asymmetry, Egger test and trim-and-fill results, this pattern is compatible with possible small-study effects, rather than indicating an independent clinical moderator. Publication year and geographic region did not clearly explain between-study heterogeneity. Overall, these findings support a conservative interpretation: the association between AAC burden and any AL appears directionally stable, but heterogeneity and possible small-study effects limit derivation of a transferable CT threshold. A complete discussion of residual uncertainty and sources of bias can be found in the “Limitations” subsection.

The biological mechanism is clinically plausible and consistent with the classical surgical model in which anastomotic integrity reflects the interaction between local technical conditions and tissue perfusion. The impact of perfusion has been validated in several studies on indocyanine green fluorescence angiography (ICG-FA), an intraoperative technique that provides real-time assessment of bowel perfusion at the intended transection and anastomotic margins. A large meta-analysis reported reduced leakage with ICG-FA in comparative studies [52], while RCT findings are context-dependent, reporting benefit in rectal cancer surgery [53] and neutral overall results in broader colorectal trial populations [54,55,56]. It should be noted, however, that ICG-FA and CT-derived vascular markers are not directly interchangeable; the first is a functional intraoperative test while the latter is a potential preoperative screening tool that may reflect impaired inflow, collateral reserve or broader vascular vulnerability. On the other hand, preoperative mesenteric stenosis grading on routine CT could serve as an opportunistic assessment of reduced inflow/collateral reserve in the form of an upstream proxy of impaired perfusion. Indeed, Arron et al. hypothesized that severe IMA disease acts as a “functional high tie,” compromising perfusion and increasing leak risk [19] and calcification-based metrics are generally interpreted in the surgical literature as markers of systemic arteriosclerosis with impaired perfusion reserve, although a direct link to anastomotic microvasculature has not been demonstrated. Imaoka et al. [57] proposed a plausible hemodynamic rationale via reduced arterial elasticity and Windkessel buffering, alongside diffuse visceral arterial disease. Moreover, adjacent evidence supporting the importance of CT-derived vascular disease markers exists for other perfusion-sensitive reconstructions [58,59,60,61]. The emerging reverse cardio-oncology literature highlights the overlap between cardiovascular disease and oncology, with studies linking atherosclerotic disease to cancer outcomes through systemic inflammatory and immune–hematopoietic pathways [62] and claims-based analyses reporting an independent association between atherosclerotic cardiovascular disease and increased risk of several cancers, including colon cancer [63]. Selected cardiovascular imaging may become relevant in complex oncologic patients; in colorectal cohorts, Gunji et al. [27] reported routine preoperative echocardiography and electrocardiography in surgical patients older than 65 years, while echocardiography remains useful as a first-line examination for suspected malignant cardiac masses, of which metastasis are the most common, followed by CT and MRI for further characterization [64,65]. Taken together, these observations support the view that CT-detected vascular disease may reflect broader host vulnerability in addition to reduced perfusion reserve.

Existing reviews have addressed similar questions but differ in scope and analytic strategy. Knight et al. [66] evaluated aortic calcification across gastrointestinal resections and highlighted heterogeneity in calcification assessment and limited confounder control in colorectal cohorts, constraining colorectal-specific inference. Tong et al. [67] provided an early colorectal meta-analysis based on a small number of studies using non-uniform quantification methods. Hoek et al. [68] demonstrated that incompatible calcification scoring methods across colorectal studies limited conventional pooling and emphasized the need for standardized vascular assessment, including stenosis grading. Liu et al. [69] reported a positive association but their search included studies up to 2022, applied different effect measures (hazard ratio—HR, not standard practice for short-term binary complications) and mixed benign and malignant indications, with subgrouping. Khan et al. [70] adopted a broader “blood supply” perspective and did not meta-analyze on grounds of heterogeneity, but nevertheless proposed CT-derived vascular assessment for AL prediction and surgical planning. Differences between prior pooled estimates and the present findings likely reflect population restrictions (CRC-only vs. mixed-indication), approach to exposure data (territory and score type) and threshold derivation. In our analyses, studies using ROC/Youden thresholds tended to yield larger effects (Figure 5), consistent with our bias diagnostics (Appendix D). Compared to prior reviews, the present work aimed to reduce statistical noise by restricting eligibility to histologically confirmed CRC with primary anastomosis, employed an updated evidence base through 2026, extended outcomes to clinically severe (grade C/ISREC-equivalent) leak and major morbidity and assessed calcification alongside the limited CRC-specific evidence on stenosis within a formal risk-of-bias and GRADE framework.

### 4.1. Strengths and Limitations

This review had several strengths, including prospective PROSPERO registration, PRISMA-based methodology, a broad search strategy, duplicate screening and extraction, formal risk-of-bias and GRADE assessments and a clinically focused population of colorectal cancer patients undergoing resection with primary anastomosis. In contrast to broader previous reviews, we evaluated CT-derived vascular disease markers specifically in relation to early post-operative outcomes after colorectal cancer surgery and considered both calcification burden and mesenteric stenosis. The main pooled association was further examined through multiple sensitivity analyses, including leave-one-out testing, trim-and-fill analysis and subgroup analyses according to cut-off derivation and measurement approach, and the direction of the main association proved stable under different sets of assumptions, although effect sizes were attenuated in some sensitivity analyses.

This study also had a number of important limitations. Firstly, the certainty rating of our conclusions was impacted by the largely retrospective and observational nature of the included studies and by incomplete adjustment for confounding variables such as baseline participant health status and intraoperative factors. Technical variables, including mechanical versus manual anastomosis, anastomotic configuration, reinforcement, number of stapler firings, anastomotic height and diversion, may influence leakage risk and may interact with impaired vascular reserve. However, these factors were inconsistently reported across the included studies and were generally unavailable as technique-specific exposure–outcome data suitable for adjustment, subgroup analysis or meta-regression. They should therefore be considered potential sources of residual confounding and heterogeneity in the present review.

The included studies were clinically and methodologically heterogeneous in both exposure and outcome definitions. CT-derived vascular disease was assessed across different arterial territories using non-uniform CT acquisition/reconstruction parameters and with different visual, semiquantitative or software-based scoring methods, while the thresholds used to define high-risk vascular disease were derived through different approaches (clinically motivated, median-based or data-driven) rather than from a prespecified standard. Outcome definitions were also variable; AL follow-up windows ranged from 30-day endpoints in several cohorts to 90-day endpoints or clinically relevant reintervention-based definitions in others, while the rest did not specify an explicit timeframe. Small-study effects and potential publication bias also remain credible concerns, as suggested by funnel plot asymmetry, Egger’s test and trim-and-fill analysis, while selective reporting of exposure specifications or outcomes cannot be excluded. In addition, because the primary meta-analyses were based on crude 2 × 2 data, pooled estimates should be interpreted as unadjusted prognostic associations rather than fully adjusted causal effects. Residual confounding therefore remains an important limitation.

Finally, restricting our analysis to histologically confirmed CRC with primary anastomosis improves clinical coherence but also reduces generalizability to broader colorectal populations. This restriction also limited the evidence for mesenteric stenosis, as only one eligible CRC-specific study provided extractable outcome data. Mixed-indication stenosis studies did not meet the inclusion criteria for the main synthesis and were therefore not incorporated into the main evidence synthesis; instead, they were retained only as contextual supplementary evidence for hypothesis-generation (Appendix E).

### 4.2. Future Directions

Future work should move from heterogeneous retrospective associations to standardized evidence of clinical decision-making. Comparisons of vascular territories relevant to colorectal perfusion, study protocols and primary scoring approach can facilitate the development of a standardized and reproducible methodology. Standardized technical operative variables should be collected together with CT-derived vascular markers to determine whether preoperative vascular disease adds predictive value beyond anastomotic technique. Prospective multicenter cohorts should validate calcification and stenosis measures using generally accepted outcome definitions and follow-up windows (ISREC Grade B/C or equivalent, and major morbidity Clavien–Dindo type ≥ III). Clinical translation could take the form of preoperative CT-based screening and risk-stratification to identify patients most likely to benefit from selective intraoperative ICG-FA confirmation at the planned transection or anastomotic site. Future validation studies could therefore evaluate integrated pathways combining standardized preoperative CT-derived vascular risk assessment with selective intraoperative ICG-FA, rather than considering the two methods as parallel and competing alternatives. The role of stenosis assessment remains understudied relative to other potential prognostic biomarkers. In conjunction with vascular anatomy, clinically significant stenosis of upstream arteries could represent a type of preoperative perfusion assessment to be used in ascertaining surgical risk. AI-enabled opportunistic CT quantification of vascular calcification, and potentially stenosis grading, may improve the reproducibility and scalability of preoperative risk assessment. Although no such AI models for surgical risk prediction currently exist, analogous applications have been developed for opportunistic cardiovascular risk prediction [71], with some studies even suggesting favorable cost-effectiveness [14]. Finally, dedicated health–economic analyses are needed to establish the utility of a CT triage-to-perfusion confirmation approach in terms of reducing the risk of clinically relevant leaks and reoperations.

## 5. Conclusions

This review supports an association between preoperative CT-derived vascular disease markers and adverse postoperative outcomes after colorectal cancer resection with primary anastomosis. Calcification-based metrics provided the most consistent associations with any AL and grade C/clinically severe AL, whereas evidence for major morbidity and stenosis-based measures remained insufficient for firm conclusions. These findings indicate that routinely acquired preoperative CT may provide opportunistic information for surgical risk stratification, but the current evidence does not establish a validated clinical decision threshold. Given the largely retrospective and methodologically variable evidence base, these findings are best viewed as a basis for prospective validation rather than directly actionable clinical thresholds. Further prospective studies using standardized CT scoring and uniform outcome definitions are needed to validate clinical benefit, derive transferable cut-off values and determine whether specific vascular territories have differential prognostic value. Clinical use should remain cautious until prospective studies validate pathways that integrate preoperative CT-based risk stratification with selective intraoperative perfusion assessment, including ICG-FA.

## Figures and Tables

**Table 1:** Summary of study characteristics.

Study	Design and Setting	Population	Tumor Location	Primary Outcome - Incidence	Secondary Outcomes ^a^	Risk of Bias (Newcastle–Ottawa Score)
Arron et al. 2022 [19]	RC and nested CC Multi-center Netherlands 2009–2018	*n* = 1273 Mean age = 67 Gender not reported	Left colon Rectum	Any AL—6% of subjects (52 cases vs. 52 controls)	-	7/9
Diao et al. 2024 [20]	RC Single-center China 2011–2020	*n* = 2412 Mean age = 62 62.4% M	Colon Rectum	Any AL—4% of subjects	Grade C AL	8/9
Eveno et al. 2016 [21]	RC Single-center France 2007–2010	*n* = 60 Mean age = 70.5 50% M/F	Left colon Rectum	Any AL—21.6% of subjects	Major complications (C-D grade ≥III) Mortality	7/9
Gunji et al. 2022 [27]	PC Single-center Japan 2017–2020	*n* = 124 Mean age = 75.3 61.3% M	Colon Rectum	Major complications (C-D grade ≥ III)—11.3% of subjects	Major complications (C-D grade ≥III) Mortality	6/9
Knight et al. 2021 [28]	RC Single-center United Kingdom 2008–2016	*n* = 657 Mean age = 68 56% M	Colon Rectum	Infective (28%) vs. non-infective (20%) complications	All complications Major complications (C-D grade ≥III) Any AL (5.3%)	8/9
Lee et al. 2020 [29]	RC Single-center South Korea 2013–2015	*n* = 583 Mean age = 66 68.8% M	Rectum	Grade C AL—5.1% of subjects	Any AL (6.3%)	6/9
Morita et al. 2022 [30]	RC Single-center Japan 2014–2016	*n* = 98 Mean age = 69 56% M	Colon Rectum	Any AL—16.3% of subjects	-	6/9
Namba et al. 2021 [31]	RC Single-center Japan 2015–2020	*n* = 147 Mean age = 73.4 55.5% M	Colon Rectum	Any AL—8.16% of subjects	-	7/9
Norooz et al. 2016 [32]	PC Single-center Iran 2012–2014	*n* = 100 Mean age = 63.7 55% M	Colon Rectum	Any AL—20% of subjects	-	5/9
Pochhammer et al. 2018 [33]	PC Single-center Germany 2014–2016	*n* = 139 Mean age = 73 52.5% M	Colon Rectum	Any AL—11% of subjects	-	7/9
Postaire et al. 2018 [34]	RC Single-center France 2011–2016	*n* = 253 Mean age = 72 Gender not reported	Right colon	Any AL—9.1% of subjects	-	6/9
Shen et al. 2019 [35]	RC Single-center China 2009–2017	*n* = 423 Mean age = 64 62.4% M	Rectum	Any AL—7.8% of subjects	Grade C AL	6/9
Turhan et al. 2025 [36]	RC Single-center Turkey 2020–2023	*n* = 151 Mean age = 65.4 61.6% M	Colon Rectum	Any AL—5.96% of subjects	-	7/9
Zhang et al. 2023 [37]	RC Single-center China 2018–2021	*n* = 292 Mean age = 67 57.7% M	Colon Rectum	AL—12.67% of subjects	-	6/9

* RC—retrospective cohort; PC—prospective cohort; CC—case–control; C-D—Clavien–Dindo. ^a^ The incidence of AL is provided in parentheses.

**Table 2:** Summary of the main findings and their corresponding level of certainty according to the GRADE criteria.

Outcome (Exposure)	Assumed Risk Without Substantial Vascular Disease	Assumed Risk with Substantial Vascular Disease Present	Relative Effect (OR, 95% CI)	Participants (Studies)	Certainty (GRADE)	Reasoning
**Major morbidity (AAC score)**	~89 per 1000	177 per 1000 (95% CI 76.54–408.51)	1.99 (95% CI 0.86–4.59)	841 (*k* = 3)	⊕◯◯◯Very low	Downgraded for ROB and imprecision ^a^.
**Any anastomotic leak (AAC score)**	~40 per 1000	123 per 1000 (95% CI 83.6–181.6)	3.08 (95% CI 2.09–4.54)	5005 (*k* = 11)	⊕⊕◯◯Low	Downgraded for ROB ^a^. Publication bias was suspected and retained as a residual concern ^b^. Sensitivity analyses informed the decision not to downgrade further for inconsistency or imprecision. Upgraded for large magnitude of effect ^c^.
**Grade C anastomotic leak (AAC score)**	~14 per 1000	37 per 1000 (95% CI 14.42–97.58)	2.68 (95% CI 1.03–6.97)	3418 (*k* = 3)	⊕◯◯◯Very low	Downgraded for ROB ^a^.
**Mortality** **(AAC score) ^d^**	Not estimable	Not estimable	Not pooled	Vascular exposure mortality data available from two AAC studies, *n* = 184	⊕◯◯◯Very low	Downgraded for ROB, indirectness from inconsistent reporting and very serious imprecision. Deaths were rare and mortality not consistently stratified by CT vascular exposure.
**Any anastomotic leak (clinically significant IMA stenosis) ^e^**	Not estimable ^f^	Not estimable	13.68 (95% CI 1.69–110.4)	104 (*k* = 1)	⊕◯◯◯Very low	Evidence came from one CRC-specific case–control analysis. Downgraded for ROB and very serious imprecision.

* Abbreviations: AAC—abdominal aortic calcification, GRADE—Grading of Recommendations Assessment, Development and Evaluation, IMA—inferior mesenteric artery, ROB—risk of bias. ^a^ Risk of bias due to observational and largely retrospective evidence, incomplete confounder adjustment and likely unaccounted residual confounding. ^b^ Publication bias was suspected in the “any anastomotic leak” outcome category and retained as a concern; however, because trim-and-fill attenuation did not eliminate the association, no additional formal downgrade was applied. ^c^ Upgrading was based on large magnitude of effect. Sensitivity analyses were considered when judging inconsistency and imprecision but were not treated as a formal GRADE upgrading domain. ^d^ Mortality was summarized narratively because death events were sparse, inconsistently defined and not consistently stratified by CT vascular exposure. ^e^ The stenosis certainty rating was based on the only eligible CRC-specific study. Mixed indication stenosis studies served as contextual supplementary evidence and were not incorporated into the main GRADE assessment. ^f^ Absolute risks were not directly estimable because the only CRC-specific study used a matched case–control analysis.

**Figure A1:**
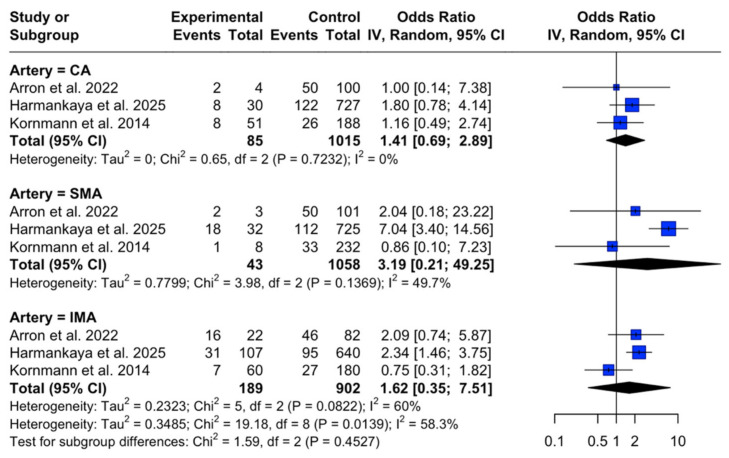
Forest plot of the association between preoperative mesenteric arterial stenosis (≥50% vs. <50%) and anastomotic leak (AL), stratified by artery (celiac axis [CA], superior mesenteric artery [SMA], inferior mesenteric artery [IMA]). These studies did not meet the CRC-specific eligibility criteria for the main synthesis unless CRC-only data were available. Results from this plot were not used for the GRADE certainty rating or for the main conclusions [17,18,19]. Squares represent individual study effects (size proportional to study weight) and diamonds represent pooled effects; horizontal lines indicate 95% CIs.

## Data Availability

All analyzed data found in this review are accessible through the referenced papers; the generated spreadsheets and code used for statistical analysis are available upon request from the corresponding author.

## Supplementary Materials

The following supporting information can be downloaded at: https://www.mdpi.com/article/10.3390/diagnostics16101449/s1, Table S1: PRISMA 2020 checklist. Ref. [72] is cited in Supplementary Materials.

## Abbreviations

The following abbreviations are used in this manuscript:

AAC	Abdominal aortic calcification
ACI	Aorto-iliac calcification index
ACS	Abdominal aortic calcification score
AL	Anastomotic leakage
BMI	Body mass index
CA	Celiac artery
CC	Case–control
CAVI	Cardio-ankle vascular index
C-D	Clavien–Dindo (classification)
CECT	Contrast-enhanced computed tomography
CI	Confidence interval
CORREA	ESCP CORREA 2022 audit
CRC	Colorectal cancer
CT	Computed tomography
ESCP	European Society of Coloproctology
GLOBOCAN	Global Cancer Observatory database
GRADE	Grading of Recommendations Assessment, Development and Evaluation
ICG-FA	Indocyanine green fluorescence angiography
IMA	Inferior mesenteric artery
I^2^	I-squared heterogeneity statistic
ISREC	International Study Group of Rectal Cancer
MEDLINE	Medical Literature Analysis and Retrieval System Online
MRI	Magnetic resonance imaging
NOS	Newcastle–Ottawa Scale
OR	Odds ratio
PC	Prospective cohort
PCS	Prospective cross-sectional
PEO	Patient, exposure, outcome
PET	Positron emission tomography
PRISMA	Preferred Reporting Items for Systematic Reviews and Meta-Analyses
PROSPERO	International Prospective Register of Systematic Reviews
RC	Retrospective cohort
RCT	Randomized controlled trial
ROB	Risk of bias
ROC	Receiver operating characteristic
SMA	Superior mesenteric artery
WoS	Web of Science Core Collection
τ^2^	Between-study variance (tau-squared)

## Appendix A. Search Strategy

**Table A1:** Full search strategy in PubMed.

Block	String	Number of Results
Cancer—1	“Colorectal Neoplasms” [mh] OR “Colonic Neoplasms” [mh] OR “Rectal Neoplasms” [mh] OR “Cecal Neoplasms” [mh] OR ((“Intestine, Large” [mh] OR “Cecum” [mh] OR “Colon” [mh] OR “Rectum” [mh] OR colorectal * [tiab] OR colon * [tiab] OR rectal * [tiab] OR rectum [tiab] OR anal [tiab] OR anus [tiab] OR appendi * [tiab] OR cecum * [tiab] OR coecum * [tiab] OR caecum * [tiab] OR cecal * [tiab] OR coecal * [tiab] OR caecal * [tiab] OR sigmoid * [tiab] OR colorectal [tiab] OR colon [tiab] OR colonic [tiab] OR rectal [tiab] OR rectum [tiab] OR “large bowel” [tiab] OR CRC [tiab]) AND (cancer * [tiab] OR carcinoma * [tiab] OR tumor * [tiab] OR tumour * [tiab] OR malignan * [tiab] OR “bowel cancer” [tiab] OR neoplas * [tiab] OR tumour * [tiab] OR cancer * [tiab] OR oncolog * [tiab] OR adenoma * [tiab] OR malignan * [tiab])	502,433 results
Surgery—2	“Colorectal Surgery” [mh] OR “colorectal surg *” [tiab] OR “colorectal resect *” [tiab] OR “hemicolectomy” [tiab] OR “colon resect *” [tiab] OR “colorectal anastomos *” [tiab] OR “LAR surgery” [tiab] OR “low anterior resection” [tiab] OR “abdominoperineal resec t*” [tiab] OR “abdominoperineal surg *” [tiab] OR “bowel obstruction surg *” [tiab] OR “proctocolectomy” [tiab] OR “ileocolectomy” [tiab] OR “sigmoidectomy” [tiab] OR “colectomy *” [tiab] OR “proctectomy” [tiab] OR “left colectomy” [tiab] OR “right colectomy” [tiab] OR “laparoscopic colectomy” [tiab] OR “colon surg *” [tiab] OR “rectal surg *” [tiab] OR “proctology *” [tiab] OR “coloproctotom *” [tiab] OR “proctocolonic” [tiab] OR ((“operat *” [tiab] OR “surg *” [tiab] OR “resect *” [tiab]) AND (“Intestine, Large” [mh] OR “Cecum” [mh] OR “Colon” [mh] OR “Rectum” [mh] OR colo * [tiab] OR recta * [tiab] OR rectu * [tiab] OR anal [tiab] OR anus [tiab] OR appendi * [tiab] OR cecum * [tiab] OR coecum * [tiab] OR caecum * [tiab] OR cecal * [tiab] OR coecal * [tiab] OR caecal * [tiab] OR sigmoid * [tiab])	276,803 results
Imaging—3	“Diagnostic Imaging” [mh] OR “Tomography, X-Ray Computed” [mh] OR “Computed Tomography Angiography” [mh] OR “CT scan *” [tiab] OR “CAT scan *” [tiab] OR “compute * tomograph*” [tiab] OR “X-ray comput *” [tiab] OR CT [tiab] OR CTA [tiab] OR “CT perfusion” [tiab] OR “perfusion CT” [tiab] OR CTP [tiab] OR “computed tomograph * angiograph*” [tiab] OR “spiral CT” [tiab] OR “helical CT” [tiab] OR “multidetector CT” [tiab] OR “dual-energy CT” [tiab]	3,460,819 results
Intervention—I	“Vascular Calcification” [mh] OR “Atherosclerosis/diagnostic imaging” [mh] OR “Mesenteric Arteries/diagnostic imaging” [mh] OR “Aorta, Abdominal/diagnostic imaging” [mh] OR “Iliac Artery/diagnostic imaging” [mh] OR radiomic * [tiab] OR “radiomic signature *” [tiab] OR “texture analysis” [tiab] OR “imaging biomarker *” [tiab] OR “imaging feature *” [tiab] OR calcif * [tiab] OR calcific * [tiab] OR “calcification” [tiab] OR “calcifications” [tiab] OR “calcified plaque” [tiab] OR “calcium score” [tiab] OR “calcium scoring” [tiab] OR “Agatston score” [tiab] OR Agatston [tiab] OR “aortic calcification” [tiab] OR “abdominal aortic calcification” [tiab] OR “iliac calcification” [tiab] OR “vascular calcification” [tiab] OR stenos * [tiab] OR stenosis [tiab] OR stenoses [tiab] OR occlus * [tiab] OR “occlusion” [tiab] OR “occlusive disease” [tiab] OR “arterial stenosis” [tiab] OR “arterial occlusion” [tiab] OR “arterial obstruction” [tiab] OR “mesenteric stenosis” [tiab] OR “mesenteric occlusion” [tiab] OR “mesenteric ischemia” [tiab] OR SMA [tiab] OR IMA [tiab] OR perfus * [tiab] OR “blood flow” [tiab] OR “tissue perfusion” [tiab] OR microperfusion [tiab] OR “CT perfusion” [tiab] OR “perfusion CT” [tiab] OR CTP [tiab]	1,018,018 results
Outcome—O	post-operat * [tiab] OR postoperat * [tiab] OR surger * [tiab] OR surgical * [tiab] OR operat * [tiab]) AND (complication * [tiab]) OR “Postoperative Complications” [mh] OR “Anastomotic Leak” [mh] OR “Anastomosis, Surgical/adverse effects” [mh] OR “Surgical Wound Infection” [mh] OR “Surgical Wound Dehiscence” [mh] OR “Ileus” [mh] OR “Mortality” [mh] OR “Reoperation” [mh] OR “Patient readmission” [mh] OR infect * [tiab] OR outcome * [tiab] OR leak * [tiab] OR hernia * [tiab] OR complicat * [tiab] OR readmi * [tiab] OR prognos * [tiab] OR predict * [tiab] OR abcess [tiab] OR abscess [tiab] OR impact [tiab] OR morbidity [tiab] OR mortality [tiab] OR adverse [tiab] OR recur * [tiab] OR recover * [tiab] OR emergen * [tiab] OR urgen * [tiab] OR death [tiab] OR survival [tiab] OR “acute kidney injury” [tiab] OR AKI [tiab] OR risk [tiab] OR peritonit * [tiab] OR adhesion [tiab] OR ischemia * [tiab] OR perforat * [tiab] OR repair [tiab] OR anastomo * [tiab] OR bleeding [tiab] OR stricture * [tiab] OR stenos * [tiab] OR “postoperative outcome *” [tiab] OR “surgical outcome *” [tiab] OR “treatment outcome *” [tiab] OR “postoperative mortality” [tiab] OR “anastomotic leak” [tiab] OR “anastomotic leakage” [tiab] OR “anastomotic failure” [tiab] OR “anastomotic dehiscence” [tiab] OR “anastomotic insufficiency” [tiab] OR “anastomotic fistula” [tiab] OR “surgical site infection” [tiab] OR SSI [tiab] OR “wound infection” [tiab] OR “intra-abdominal infection” [tiab] OR abscess* [tiab] OR ileus [tiab] OR “postoperative ileus” [tiab] OR “paralytic ileus” [tiab] OR “organ dysfunction” [tiab] OR “organ failure” [tiab] OR “multiple organ dysfunction” [tiab] OR sepsis [tiab] OR reoperate * [tiab] OR “re-operation” [tiab] OR readmiss * [tiab] OR “hospital readmission” [tiab] OR “Clavien-Dindo” [tiab] OR Clavien [tiab] OR “complication grade” [tiab]	14,872,887 results
Population—P	1 *AND* 2 *AND* 3	126,080 results
Total	P *AND* I *AND* O	1717 results
Total—study exclusion	Total NOT (Review [Publication Type] OR “Case reports” [Publication Type]) NOT (animals [Mesh] NOT humans [Mesh])	1067 results

**Table A2:** Full search strategy in Google Scholar. Retrieval of Google Scholar search results was automated using Publish or Perish (Harzing, A.W. [2007] Publish or Perish, available from https://harzing.com/resources/publish-or-perish [accessed on 1 January 2026]).

String	Results Screened
“abdominal aortic calcification” CT colorectal anastomotic leak	72
“aortic calcification index” CT rectal anastomotic leak	20
“aortoiliac calcification” CT rectal surgery anastomotic leak	29
“iliac artery calcification” CT rectal resection anastomotic leak	9
“calcium score” aortic CT colorectal anastomotic leak	93
Agatston CT rectal cancer surgery anastomotic leak	71
“vascular calcification” CT colorectal surgery	113
“inferior mesenteric artery” stenosis CT rectal anastomotic leak	200
“inferior mesenteric artery” occlusion CT rectal anastomotic leak	200
“superior mesenteric artery” stenosis CT right colectomy anastomotic leak	200
“celiac trunk” stenosis CT right colectomy anastomotic leak	200
“mesenteric artery stenosis” CT colorectal surgery complication	149
“mesenteric occlusive disease” CT colorectal anastomotic leak	28

## Appendix B. Newcastle–Ottawa Scores

**Table A3:** NOS scoring of the selected studies for each category. Each ☆ denotes one Newcastle-Ottawa Scale point awarded for the corresponding item.

Study	Selection				Comparability		Outcome/Exposure			Total 0–9	Study Quality
	S1	S2	S3	S4	C1	C2	O1/E1	O2/E2	O3/E3		
Arron et al. 2022 [19]	☆	☆	☆	☆	☆		☆	☆		7	Good
Diao et al. 2024 [20]	☆	☆	☆	☆	☆	☆	☆	☆		8	Good
Eveno et al. 2016 [21]	☆	☆	☆	☆			☆	☆	☆	7	Good
Gunji et al. 2022 [27]	☆	☆	☆	☆	☆		☆			6	Fair
Knight et al. 2021 [28]	☆	☆	☆	☆	☆	☆	☆	☆		8	Good
Lee et al. 2020 [29]	☆	☆	☆	☆	☆		☆			6	Fair
Morita et al. 2022 [30]	☆	☆	☆	☆			☆	☆		6	Fair
Namba et al. 2021 [31]	☆	☆	☆	☆	☆		☆	☆		7	Good
Norooz et al. 2016 [32]	☆	☆	☆	☆			☆			5	Fair
Pochhammer et al. 2018 [33]	☆	☆	☆	☆	☆		☆	☆		7	Good
Postaire et al. 2018 [34]	☆	☆	☆	☆			☆	☆		6	Fair
Shen et al. 2019 [35]	☆	☆	☆	☆	☆		☆			6	Fair
Turhan et al. 2025 [36]	☆	☆	☆	☆	☆	☆	☆			7	Good
Zhang et al. 2023 [37]	☆	☆	☆	☆	☆		☆			6	Fair

Items: Selection: S1 representativeness; S2 non-exposed from same cohort; S3 exposure ascertained from records/CT; S4 outcome absent at baseline. Comparability: C1 key confounders; C2 additional confounders. Outcome: O1 outcome assessment; O2 follow-up long enough; O3 adequacy of follow-up. Exposure: E1 ascertainment of exposure; E2 same method for cases/controls; E3 non-response.

## Appendix C. Adjusted Analyses

**Table A4:** Summary of confounder-control strategies and adjusted (or matched) estimates reported in included studies.

Study	Outcome	CT Vascular Marker (Cut Point)	Adjustment/Matching (Covariates)	Adjusted/Matched Estimate
Arron et al. 2022 [19]	Any AL	IMA stenosis ≥ 70% or occlusion	Matched CC (1:1) on age, BMI, cardiovascular comorbidity	Clinically significant IMA stenosis: 21.2% (AL cases) vs. 1.9% (controls); *p* = 0.01
Diao et al. 2024 [20]	Any AL	AAC (present vs. absent)	Multivariable logistic regression; sex, HTN, T2DM, tumor location, tumor stage, smoking, drinking	OR 1.443 (95% CI 0.871–2.358); *p* = 0.157
Gunji et al. 2022 [27]	Major complications (C-D ≥ III)	AAC score (continuous variable)	Multivariable logistic regression; covariates (*p* < 0.25 in univariate): sex, ASA ≥ 3, hyperlipidemia, chronic renal failure, stage, CAVI, laparoscopic surgery, anterior resection, operative time, AAC score	OR 1.083 (95% CI 1.009–1.163); *p* = 0.026
Knight et al. 2021 [28]	AL Major complications (C-D III–V)	Distal aortic calcification (None/Minor/Major)	Multivariable logistic regression; covariates: significant univariate predictors (sex, tumor site, surgical approach, smoking)	Any complications: OR 1.05 (95% CI 0.83–1.33); *p* = 0.70. Major complications: OR 1.32 (95% CI 0.90–1.94); *p* = 0.15
Lee et al. 2020 [29]	Grade C AL	Aortoiliac calcification score ≥ 3 vs. <3	Backward stepwise logistic regression; sex, ASA score, operative time ≥ 180 min, AICS ≥ 3	OR 2.677 (95% CI 1.040–6.890); *p* = 0.041
Namba et al. 2021 [31]	AL	Agatston score (high vs. low)	Multivariable logistic regression; male sex, lower rectal tumor, preop WBC, Agatston score	OR 6.09 (95% CI 1.48–25.0); *p* = 0.012
Pochhammer et al. 2018 [33]	AL	Internal iliac artery calcification (volume score ≥ 30 vs. <30; ROC-derived)	Logistic regression; multivariable model retained internal iliac volume score + renal disease	Multivariable: calcification not independent (*p* = 0.08); renal disease *p* = 0.001 ^a^.
Shen et al. 2019 [35]	AL	Aortic Calcification Index (ACI) > 4.8%	Multivariable logistic regression (after LASSO selection); CAD, TG > 1.7, glucose > 6.1, UICC stage III-IV, op time > 240 min, diverting ileostomy, ACI	OR 2.391 (95% CI 1.040–5.494); *p* = 0.040
Turhan et al. 2025 [36]	AL	Aortic calcification >50%	Multivariable logistic regression; TNM stage, HTN, cardiovascular disease, neoadjuvant therapy	OR 10.38 (95% CI 1.243–92.118); *p* = 0.032
Zhang et al. 2023 [37]	AL	SMA calcium volume score (continuous variable)	Multivariable logistic regression; tumor location, albumin, lymphocyte count, NLR, SMA calcium volume score	OR 6.810 (95% CI 1.870–24.801); *p* = 0.004

* Abbreviations: AAC—abdominal aortic calcification; AC—aortic calcification; ACI—aortic calcification index; AICS—aortoiliac calcification score; AL—anastomotic leak; ASA—American Society of Anesthesiologists; BMI—body mass index; CAD—coronary artery disease; CAVI—cardio-ankle vascular index; C-D—Clavien–Dindo; HTN—hypertension; IMA—inferior mesenteric artery; NLR—neutrophil-to-lymphocyte ratio; SMA—superior mesenteric artery; T2DM—type 2 diabetes mellitus. ^a^ Univariate internal iliac Ca score OR 1.005 (95% CI 1.001–1.008; *p* = 0.01).

## Appendix D. Sensitivity Analysis

**Table A5:** Results of the sensitivity analysis for the different harmonization assumptions (categorical moderator univariate meta-regression).

Moderator (Comparison)	k (Ref/Comp)	Test of Moderator	*p*-Value	Exp(β) (Ratio of ORs) (95% CI for OR Exp(β))	Heterogeneity (Residual τ^2^; R^2^)
Cut point type (ROC-derived vs. non-ROC-derived) ^a^	7/4	5.42	0.044	2.32 (1.02–5.24)	0.0217/82.5%
Exposure measurement (visual vs. quantitative) ^b^	5/6	2.07	0.184	0.61 (0.28–1.33)	0.82/34%
Vascular territory (non-Aorta-only vs. Aorta-only) ^c^	6/5	0.026	0.875	1.06 (0.46–2.45)	0.16/0%
NOS score (good vs. fair)	6/5	0.0055	0.942	0.97 (0.42–2.25)	0.16/0%

^a^ ROC-derived thresholds were obtained through ROC/Youden optimization; non-ROC-derived thresholds were prespecified (e.g., median-based cut point or ordinal staging). ^b^ Visual scores include binary presence/absence and visual ordinal staging (e.g., none/minor/major); quantitative measurements are software-derived numerical metrics. ^c^ Aorta-only scores include the whole abdominal aorta or infrarenal aorta only; non-Aorta-only scores include aortoiliac, splanchnic-only (celiac and mesenteric arteries), or mixed aortic–splanchnic territories. F, test of moderators from the mixed-effects meta-regression; exp(β) is the multiplicative difference in pooled OR between the compared categories as coded in the model.

**Table A6:** Estimator comparison check between the REML + Hartung–Knapp and the DerSimonian–Laird methods for CI estimation.

Outcome/ Exposure	Studies/ Participants	Primary Model: REML + Hartung–Knapp OR (95% CI)	Primary Model: DerSimonian–Laird OR (95% CI)	Effect of Estimator Choice
Major morbidity/AAC	*k* = 3/*n* = 841	1.99 (0.86–4.59)	1.99 (1.27–3.11)	DerSimonian–Laird produced a narrower CI and changed nominal statistical significance
Any anastomotic leak/AAC	*k* = 11/*n* = 5005	3.08 (2.09–4.54)	3.04 (2.15–4.31)	DerSimonian–Laird produced a narrower CI without changing statistical significance
Grade C anastomotic leak/AAC	*k* = 3/*n* = 3418	2.68 (1.03–6.97)	2.68 (1.59–4.52)	DerSimonian–Laird produced a narrower CI without changing statistical significance

Abbreviations: AAC, abdominal aortic calcification; CI, confidence interval; OR, odds ratio; REML, restricted maximum likelihood.

**Table A7:** Exploratory univariable meta-regression for the association between abdominal aortic calcification burden and any anastomotic leak.

Moderator	Studies Included	Coding	Ratio of ORs	95% CI	*p*-Value	Residual τ^2^ Residual I^2^ R^2^	Interpretation
Sample size	11	Per 100-participant increase	0.963	0.932–0.994	0.024	0.0187 6.65% 84.9%	Larger studies reported smaller effect estimates consistent with small study effects
Publication year	11	Per additional calendar year within the observed range	0.94	0.801–1.103	0.402	0.1009 30.07% 18.7%	Publication year did not clearly explain heterogeneity
Geographic region	11	Asia vs. Europe	1.488	0.668–3.310	0.290	0.1085 32.81% 12.7%	No clear regional effect modification was observed

## Appendix F. Domain-Level GRADE Evidence Decision Table

**Table A8:** Domain-level GRADE evidence decision table. The table should be read row-wise. For each outcome, the contributing evidence base and effects are shown first, followed by the starting certainty and the domain-level GRADE judgements that determined the final certainty rating. Downgrades and upgrades are indicated in the relevant domain columns; the explanation line below each outcome summarizes the rationale for the final rating.

Starting Certainty	Risk of Bias	Inconsistency	Indirectness	Imprecision	Publication Bias	Upgrade Factors	Final Certainty
Major morbidity/abdominal aortic calcification score *k* = 3/*n* = 841 OR 1.99, 95% CI 0.86–4.59							
Low	Serious; downgraded one level	Not serious	Not serious	Serious; downgraded one level	Not assessable	None	⊕◯◯◯ Very low
Explanation:	Low starting certainty because the evidence was based on observational studies. ROB was judged serious because of retrospective designs and incomplete confounder control. Serious imprecision on account of the CI crossing the null and including both no important effect and clinically important harm.						
Any anastomotic leak/abdominal aortic calcification score *k* = 11/*n* = 5005 OR 3.08, 95% CI 2.09–4.54							
Low	Serious; downgraded one level	Not serious	Not serious	Not serious	Suspected; concern but no formal downgrade	Large magnitude of effect; upgraded one level	⊕⊕◯◯ Low
Explanation:	Low starting certainty because the evidence was based on observational studies. ROB was judged serious because most studies were retrospective and incompletely adjusted. Moderate heterogeneity but leave-one-out and sensitivity analyses preserved the direction of effect. Publication bias was suspected, but, since trim-and-fill did not eliminate the association, no additional formal downgrade was applied. Upgraded for large magnitude of effect.						
Grade C anastomotic leak/abdominal aortic calcification score *k* = 3/*n* = 3418 OR 2.68, 95% CI 1.03–6.97							
Low	Serious; downgraded one level	Not serious	Not serious	Not serious	Not assessable	None	⊕◯◯◯ Very low
Explanation:	Low starting certainty because the evidence was based on observational studies. Serious ROB because of retrospective and incompletely adjusted evidence.						
Mortality/abdominal aortic calcification score *k* = 2/*n* = 184; additional deaths mentioned without exposure-stratified data Not pooled/not estimable							
Low	Serious; downgraded one level	Not reliably assessable	Serious; downgraded one level	Serious; downgraded one level	Not assessable	None	⊕◯◯◯ Very low
Explanation:	Effect estimate not pooled; downgraded for serious imprecision because mortality events were sparsely reported and some studies did not stratify mortality by exposure, hindering the extraction of stable mortality estimates. Downgraded for indirectness because post-operative and all-cause mortality were not separated. Only two AAC studies provided mortality information compatible with vascular exposure assessment, while additional studies reported deaths without stratification by calcium score category or without perioperative timing.						
Any anastomotic leak/clinically significant IMA stenosis *k* = 1/*n* = 104 OR 13.68, 95% CI 1.69–110.4							
Low	Serious; downgraded one level	Not assessable; single-study	Not serious	Very serious; downgraded two levels	Not assessable	None	⊕◯◯◯ Very low
Explanation:	Evidence came from a single CRC-specific matched case–control study. The association was large, but the estimate was very imprecise with a wide CI and few exposed events. Inconsistency and publication bias could not be assessed. No upgrade was applied because the evidence was single-study and the association was imprecise. Mixed-indication stenosis studies were not used to determine this certainty rating.						

Abbreviations: AAC, abdominal aortic calcification; CI, confidence interval; CRC, colorectal cancer; GRADE, Grading of Recommendations Assessment, Development and Evaluation; IMA, inferior mesenteric artery; OR, odds ratio; ROB, risk of bias.
